# Is There Evidence for a Rostral-Caudal Gradient in Fronto-Striatal Loops and What Role Does Dopamine Play?

**DOI:** 10.3389/fnins.2018.00242

**Published:** 2018-04-12

**Authors:** David A. Vogelsang, Mark D'Esposito

**Affiliations:** ^1^Helen Wills Neuroscience Institute, University of California, Berkeley, Berkeley, CA, United States; ^2^Department of Psychology, University of California, Berkeley, Berkeley, CA, United States

**Keywords:** lateral prefrontal cortex, hierarchical processing, fronto-striatal loops, dopamine, receptor distribution

## Abstract

Research has shown that the lateral prefrontal cortex (LPFC) may be hierarchically organized along a rostral-caudal functional gradient such that control processing becomes progressively more abstract from caudal to rostral frontal regions. Here, we briefly review the most recent functional MRI, neuropsychological, and electrophysiological evidence in support of a hierarchical LPFC organization. We extend these observations by discussing how such a rostral-caudal gradient may also exist in the striatum and how the dopaminergic system may play an important role in the hierarchical organization of fronto-striatal loops. There is evidence indicating that a rostral-caudal gradient of dopamine receptor density may exist in both frontal and striatal regions. Here we formulate the hypothesis that dopamine may be an important neuromodulator in hierarchical processing, whereby frontal and striatal regions that have higher dopamine receptor density may have a larger influence over regions that exhibit lower dopamine receptor density. We conclude by highlighting directions for future research that will help elucidating the role dopamine might play in hierarchical frontal-striatal interactions.

## Introduction

Cognitive control refers to the ability to select and regulate thoughts and actions that are in accordance with our internal behavioral goals or intentions (Braver, 2012). Cognitive control processes are likely organized in a hierarchical fashion such that higher level representations can influence the processing of lower level representations (Badre, 2008). The lateral prefrontal cortex (LPFC) is a core region in the network of brain areas that are important for cognitive control, and several studies have indicated that different types of cognitive control processes may be supported by distinct subregions within the PFC (Badre and D'Esposito, 2009). One characteristic feature of the LPFC function is the ability to actively maintain representations of rules and goals in order to provide a top-down influence over perception and action systems, which can facilitate processing of relevant information necessary for guiding behavior (Miller and Cohen, 2001; Miller and D'Esposito, 2005). Although significant theoretical and experimental progress has been made toward understanding PFC organization, the precise functional architecture of cognitive control still remains a subject of debate.

One organizational scheme of LPFC that has gained significant empirical support proposes a hierarchical organization along a rostral-caudal gradient whereby processing becomes progressively abstract from caudal to rostral LPFC areas (Koechlin et al., 2003; Ramnani and Owen, 2004; Koechlin and Summerfield, 2007; Badre, 2008; Botvinick, 2008; Badre and D'Esposito, 2009). Support for this functional organization is derived from functional MRI (fMRI) studies (Koechlin et al., 2003; Badre and D'Esposito, 2007; Badre et al., 2010; Nee and Brown, 2012a; Nee and D'Esposito, 2016), structural MRI studies of cortical thickness, myelination, and cell body density (Thiebaut de Schotten et al., 2016), human electrocorticography studies (Voytek et al., 2015) and human lesion studies (Badre et al., 2009; Kayser and D'Esposito, 2013; Azuar et al., 2014). In this article, we will briefly review this literature. However, our main goal is to review the evidence that a similar rostral-caudal functional gradient may also exist in the striatum via fronto-striatal loops, and that the dopaminergic system may play a critical role in this organizational scheme.

## Empirical evidence for a rostral-caudal gradient of frontal function

Different models exist regarding the functional organization of LPFC. One model proposes a hierarchical organization that is based on a “cascade” of information processing whereby cognitive control processes resolve competition of different action representations based on context (Koechlin et al., 2003). These various cognitive control processes are proposed to be hierarchically organized such that information is directed from higher to lower areas and each separate control signal is processed in distinct regions along a rostral-caudal LPFC gradient. The lowest level of control is proposed to require premotor cortex, which selects motor actions in response to specific stimuli. The mid level of control is proposed to require caudal/mid LPFC, which acts to select premotor representations according to external contextual information. The highest level of control is proposed to require rostral LPFC areas, which selects representations in caudal LPFC according to whether the stimuli are remote in time from the execution of an action (Koechlin et al., 2003; Koechlin and Summerfield, 2007). Thus, this cascade model of cognitive control proposes that as actions become increasingly more abstract, the timescale between those actions also increases.

A different model proposes that the rostral-caudal LPFC gradient is based on the concept of “policy” abstraction (Badre and D'Esposito, 2009). A policy is the relationship between the state of a system, its associated action and an anticipated outcome. For example, a simple rule would be that when you are in your office and the phone rings, you pick up the phone, and that when you are in your colleague's office and the phone rings, you do not pick up the phone (e.g., first order policy). In this example, it is the context of which office you are in (yours vs. your colleague's) that is informative regarding what rule is applicable. However, an additional, more abstract rule may also be necessary to determine the appropriate response in the current context. For example, particular colleagues may have also told you that when you are in their office and they are away, it is fine to pick up the phone when it rings to take a message for them (e.g., second order policy). Thus, policy abstraction refers to the degree to which a given goal representation forms a generalization over lower level goal representations.

Evidence in support of the policy abstraction model was obtained in an fMRI study where young participants performed four types of tasks that manipulated representational hierarchy ranging from low to high abstraction (Badre and D'Esposito, 2007). At the lowest level of abstraction, the “response” task, participants had to press a button in response to colored squares. In the next higher level, the “feature” task, participants had to make responses based on the features of the object that was presented in the context of a specific colored square (e.g., subjects have to judge about the orientation of an object and press “positive” if the color of the square was red). At the next higher level, the “dimension” task, participants were instructed to judge whether two objects presented in a color square were similar or different along a certain dimension. Importantly, the color of the square determined what kind of task participants had to perform (e.g., if the color of the square is blue you have to judge whether the two objects differ in shape; if the color is orange you have to do the texture task in which you judge whether the texture of the objects is the same or not). This dimension task requires a higher level of abstraction than the feature and response tasks because one first has to consider the context (i.e., color of the square), which determines the appropriate task (e.g., is the shape of the objects the same or not). In the highest level of abstraction, the “context” task, participants performed the dimension task, however, task abstraction was increased by varying the frequency of the task sets and color to dimension mappings (e.g., in block 1 if the color of the square is yellow you do the shape task and if it is block 2 and the color of the square is yellow you do the orientation task). This task is the most abstract of all four tasks because knowledge of the temporal context (e.g., knowing this is block 1 vs. block 2) is required to select the appropriate context that determines the specific dimension (e.g., if it is block 2, then blue colored square means you do the shape task, whereas a red colored square means you do the orientation task).

In each of the four abstraction tasks, a parametric modulation was used with three levels of complexity (low, mid and high levels of competition), and each of these was associated with a distinct stimulus-response mapping. Thus, the hierarchical representations are distinguished based on the abstractness of an action/response representation that needs to be selected. This resulted in a nested design whereby the lowest level of complexity in each abstraction task was theoretically equivalent to the mid-level of complexity in the subordinate task. The parametric modulation of complexity levels within each level of abstraction allowed for examination of frontal cortex engagement at each hierarchical level as well as ruling out the possibility that frontal activation was simply due to task difficulty rather than the abstractioness of the action representation to be selected (Badre and D'Esposito, 2007).

**Figure 1:**
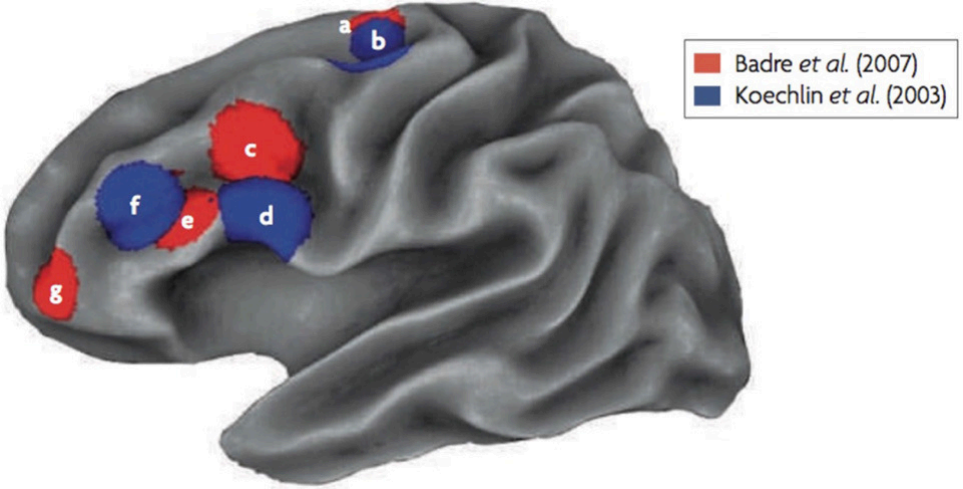
fMRI results of the Koechlin et al. (2003) and Badre and D'Esposito (2007) experiments. Regions labeled as “a” and “b” are the lowest abstraction levels (sensory and response tasks), labels “c” and “d” are second lowest in abstraction (context and feature tasks), labels “e” and “f” are the third highest in abstraction (episodic and dimension tasks) and label “g,” that was only used by Badre and D'Esposito (2007), is the highest in abstraction (context task).

A clear caudal to rostral gradient along the LPFC depending on the level of policy abstraction was found (see Figure 1; Badre and D'Esposito, 2007). For the lowest level of abstraction (i.e., response task), activity was observed in the premotor cortex. The next higher level of abstraction, the feature task, activated the pre-premotor cortex, a region rostral to the premotor cortex. For the next higher dimension task, activity was observed in the inferior frontal sulcus (likely on the border of Brodmann areas 45 and 9/46). The highest level of abstraction activated the frontopolar cortex, a rostral area in the LPFC (likely Brodmann area 10). Taken together, even though the cascade and policy abstraction models differ slightly conceptually, both studies that have tested these models provide convergent evidence for a rostral-gradient along the frontal cortex supporting cognitive control processes (see Figure 1).

Since these two studies were published, many fMRI studies have replicated these findings with different types of tasks (Nee and Brown, 2012a,b; Bahlmann et al., 2014, 2015; Nee et al., 2014; Nee and D'Esposito, 2016; for a meta-analysis see Badre and Nee, 2018). Also, a rostral-caudal gradient of function within frontal cortex is observed regardless of the stimulus domain (Bahlmann et al., 2014), and during tasks that require various levels of motivation (Bahlmann et al., 2015).

A few studies have questioned whether a rostral-caudal gradient exists in the LPFC. For example, Crittenden and Duncan (2014) propose that more rostral cortex becomes activated when a task becomes more difficult. In contrast, Reynolds et al. (2012) propose that regional differences in frontal cortex are driven by the demand to maintain context in working memory. Given that the purpose of our review is not to reconcile differences in various models of frontal cortex organization, we refer the reader to a recent review that considers these alternative explanations in detail (see Badre and Nee, 2018).

## Is the rostral-caudal gradient of function in LPFC organized hierarchically?

Two fMRI studies have suggested that frontal cortex is organized hierarchically (Koechlin et al., 2003; Nee and D'Esposito, 2016). In the first study (Koechlin et al., 2003), structural equation modeling of fMRI data was used to determine interactions between frontal regions during a task with various levels of cognitive control. During the task requiring the highest level of control (the episodic task), the most rostral frontal area (likely area 46) had a greater influence over mid/caudal-LPFC (likely area 44). For the mid-level of control (the context task), mid/caudal-LPFC influenced processing in the most caudal frontal area (around premotor cortex), whereas the lowest level of control (the sensory task) did not exert a directional influence on any frontal region. These results provide indirect evidence that frontal cortex may be organized in a hierarchical manner with more rostral regions situated at the top of the hierarchy.

In a second study (Nee and D'Esposito, 2016), dynamic causal modeling (DCM) of fMRI data was used to determine interactions between frontal regions during a task that was almost identical to that used in the Koechlin et al. (2003) study. In the task, participants were asked to attend to stimulus features (feature control, lowest level of abstraction), attend to a feature depending on the presented context (contextual control, considered to be a higher level of abstraction) and hold a stimulus in mind over the course of several trials in anticipation of a future stimulus (temporal control, considered the highest level of abstraction). The DCM analysis produced findings that differed from previous views regarding the hierarchical organization of frontal cortex that proposed that the frontopolar cortex (around area 10) is the top of the frontal hierarchy (e.g., Ramnani and Owen, 2004; Badre and D'Esposito, 2007). In contrast, DCM found that mid-LPFC had the strongest hierarchical asymmetries in connectivity, consistent with this region serving as the top of the frontal hierarchy. Also, there were top-down influences from rostral PFC to mid LPFC, and bottom-up influences from caudal LPFC to mid LPFC. These findings support a view that mid-LPFC forms a nexus where information converges to influence action (Nee and D'Esposito, 2016). This new conceptualization of frontal cortex can be reconciled with previous views. In brief, it proposes that whereas the mid-LPFC is the top of a “control” hierarchy' (which refers to feedback bias signals), the most rostral regions of frontal cortex still represent more abstract information than more caudal regions, which would place it at the top of a “representational” hierarchy (which refers to the degree of abstraction). In this way, rostral LPFC is a domain-specific region, analogous to caudal LPFC, with both areas providing convergent input into mid-LPFC (see Badre and Nee, 2018 for a more thorough description of these ideas).

The conclusions drawn by the Nee and D'Esposito (2016) study are consistent with findings by Goulas et al. (2014) who examined the hierarchical organization of the lateral frontal cortex in macaque monkeys by analyzing connectivity data from tracing studies. Specifically, they examined the hierarchical organization of the lateral frontal cortex by testing whether regions “higher” in the hierarchy have more efferent relative to afferent connections compared to regions “lower” in the hierarchy. This type of analysis provides evidence whether a particular region is influencing other regions more than vice versa. Similar to the Nee & D'Esposito human fMRI study, it was found that the mid-LPFC (around area 46) exhibited more efferent connections compared to more rostral and caudal frontal regions, suggesting that the mid-LPFC may be the top of a “control” hierarchy. It is important to note, however, that one must be cautious when drawing conclusions about human LPFC organization based on monkey anatomy. For example, it is proposed that human lateral frontopolar cortex may not have a functional homolog in monkeys, and may be uniquely human (Koechlin, 2011; Neubert et al., 2014). Also, anatomical evidence suggests that monkey frontopolar cortex (Brodmann's area 10) likely corresponds to human ventromedial prefrontal cortex (Carmichael and Price, 1996; for a review see Tsujimoto et al., 2011).

Nee and D'Esposito (2017) recently replicated and extended the findings of their fMRI study by applying continuous theta-burst stimulation (cTBS) to temporarily disrupt function in three frontal regions while participants performed the task involving three levels of a representational hierarchy. Caudal LPFC cTBS impaired performance on the task with the lowest hierarchical level, mid-LPFC cTBS impaired performance on the next highest level, and rostral LPFC cTBS impaired performance on the highest level. Together, these fMRI and cTBS findings suggest that the rostral LPFC is the top of the representational hierarchy, whereas the DCM analyses of both Nee & D'Esposito studies suggest that mid-LPFC is a critical nexus for a control hierarchy. This is a novel hypothesis that we will return to later in the review when considering the role of dopamine in the hierarchical organization of frontal cortex.

Additional indirect evidence for a hierarchical frontal cortex organization has also been reported in a human intracranial electrocorticography (ECoG) study of epileptic patients. Using the dimension and response tasks from the Badre and D'Esposito (2007) fMRI study, Voytek et al. (2015) found there was an increase in the synchrony of theta band oscillations between the LPFC and premotor/motor cortex during the performance of the task with a higher level of abstraction. Furthermore, theta-gamma phase amplitude coupling, during which the amplitude of the gamma frequency is modulated by the phase of the theta frequency, became stronger across the LPFC and premotor/motor regions as the level of abstraction increased. Additionally, there was also an effect of directionality of theta-gamma phase amplitude coupling such that theta phase in the LPFC was a stronger predictor of high gamma power in premotor/motor cortex, than that theta phase in premotor/motor cortex was of high gamma in LPFC. This finding suggests a hierarchical relationship between these regions with theta phase in the rostral frontal regions influencing gamma power in more caudal regions.

Direct evidence for a hierarchical organization of cortical regions can only be derived by a (functional) lesion method (e.g., patients with focal brain lesions or transcranial magnetic stimulation (TMS) in healthy subjects) that can provide causal evidence that one cortical area influences another area in an asymmetric way. To determine whether rostral frontal regions directly influence caudal regions, more than vice versa, Badre et al. (2009) tested 12 patients with frontal lesions on the tasks used in the Badre and D'Esposito fMRI study (Badre and D'Esposito, 2007). Specifically, they tested the hypothesis that if more rostral frontal regions influence processing of more caudal regions, then performance on tasks involving higher-order control should be impaired by damage to a region involved in lower-order processing (the pre-premotor region that supports more concrete actions engaged by the feature task), even when the higher-order region is intact. However, the reverse prediction should not hold. Following damage to a region involved in higher-order processing (inferior frontal sulcus that supports the dimension task), performance should be unaffected for tasks that only require the lower-order region (e.g., feature task). This is exactly what was observed in this study. Again, this hypothesized asymmetric pattern of deficits can only be tested with a causal method (e.g., lesions or TMS) and cannot be directly tested with neurophysiological methods. It was also reasoned that due the asymmetric dependencies of a hierarchical organization, impairments in tasks that require a higher level of abstraction are more likely to occur than impairments in tasks that require lower levels of abstraction regardless of the location of the frontal lesion. It was found that across all tasks, the probability of a task deficit was 62%. Importantly, the probability of a deficit at any task, given a deficit at a lower level, was 91%, which is significantly higher than the probability of a deficit on any task (i.e., 62%). In contrast, the probability of a deficit on any given task given the impairment at a higher level was 76%, a weak change compared to the 62% probability of a deficit on any task. These findings were replicated in another patient study by Azuar et al. (2014) who tested 26 patients with LPFC and premotor lesions on the original task used in the Koechlin fMRI study (Koechlin et al., 2003). It was found that (1) patients with premotor lesions performed significantly worse than healthy controls on all three hierarchical levels; (2) patients with mid-LPFC lesions performed worse on mid and high levels but not on the lowest level; (3) patients with rostral LPFC lesions performed worse than healthy controls on only the highest level. Together, these two patient studies provide direct evidence that frontal cortex is hierarchically organized along a rostral-caudal gradient.

## The organization of fronto-striatal loops and function

**Figure 2:**
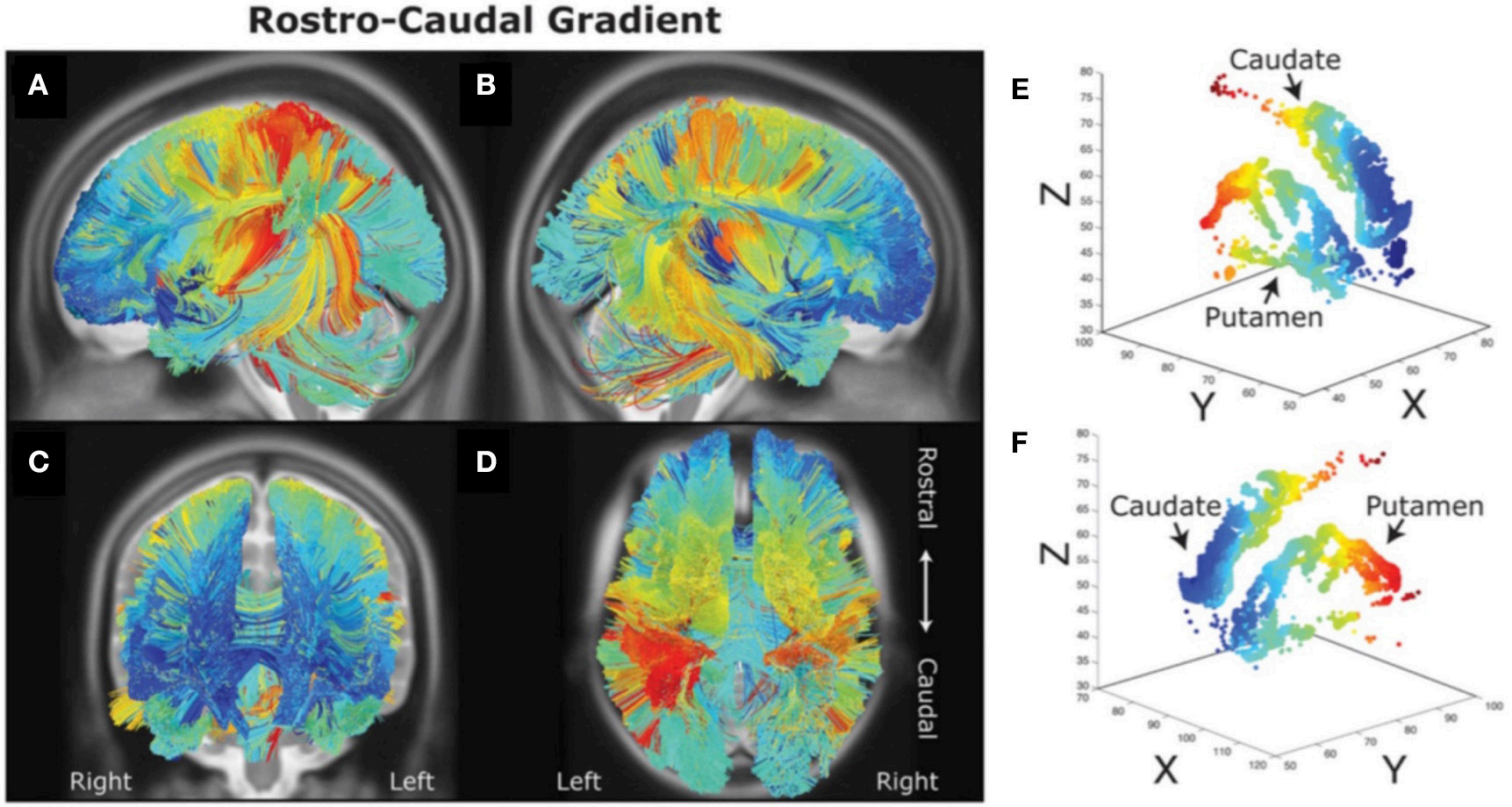
Rostral-caudal gradient of fronto-striatal connectivity **(A–F)** as measured with diffusion spectrum imaging. It can be observed that rostral (blue) and caudal (red) prefrontal areas are connected with rostral and caudal striatal areas respectively. Figure adapted from Jarbo and Verstynen (2015) with permission from the publisher.

There are distinct topographic projections from frontal, premotor and motor cortex to striatal regions (Alexander et al., 1986; Haber et al., 2000; Haber, 2004; Draganski et al., 2008). One of the first studies in humans that performed tractography on magnetic resonance diffusion spectrum imaging data found that these frontal-striatal loops may be organized along a rostral-caudal axis potentially supporting a hierarchical organization (Verstynen et al., 2012; see Figure 2). A topography was found whereby rostral LPFC projected into the rostral parts of the striatum, whereas caudal frontal areas projected into caudal parts of the striatum. Importantly, the projections from each frontal cortical area into the striatum had a degree of overlap with projections from another frontal area. Approximately 20% of lateral frontal cortex fibers projected to areas in the striatum that receives projections from other lateral frontal cortex regions, suggesting that there may be convergence of information from fronto-striatal projections. Also, a gradient in structural asymmetry in fronto-striatal connections was found whereby rostral LPFC areas connected to both rostral and caudal striatum, whereas caudal frontal areas only connected to the caudal striatum. This pattern is consistent with a rostral-caudal hierarchical organization of frontal-striatal loops.

**Figure 3:**
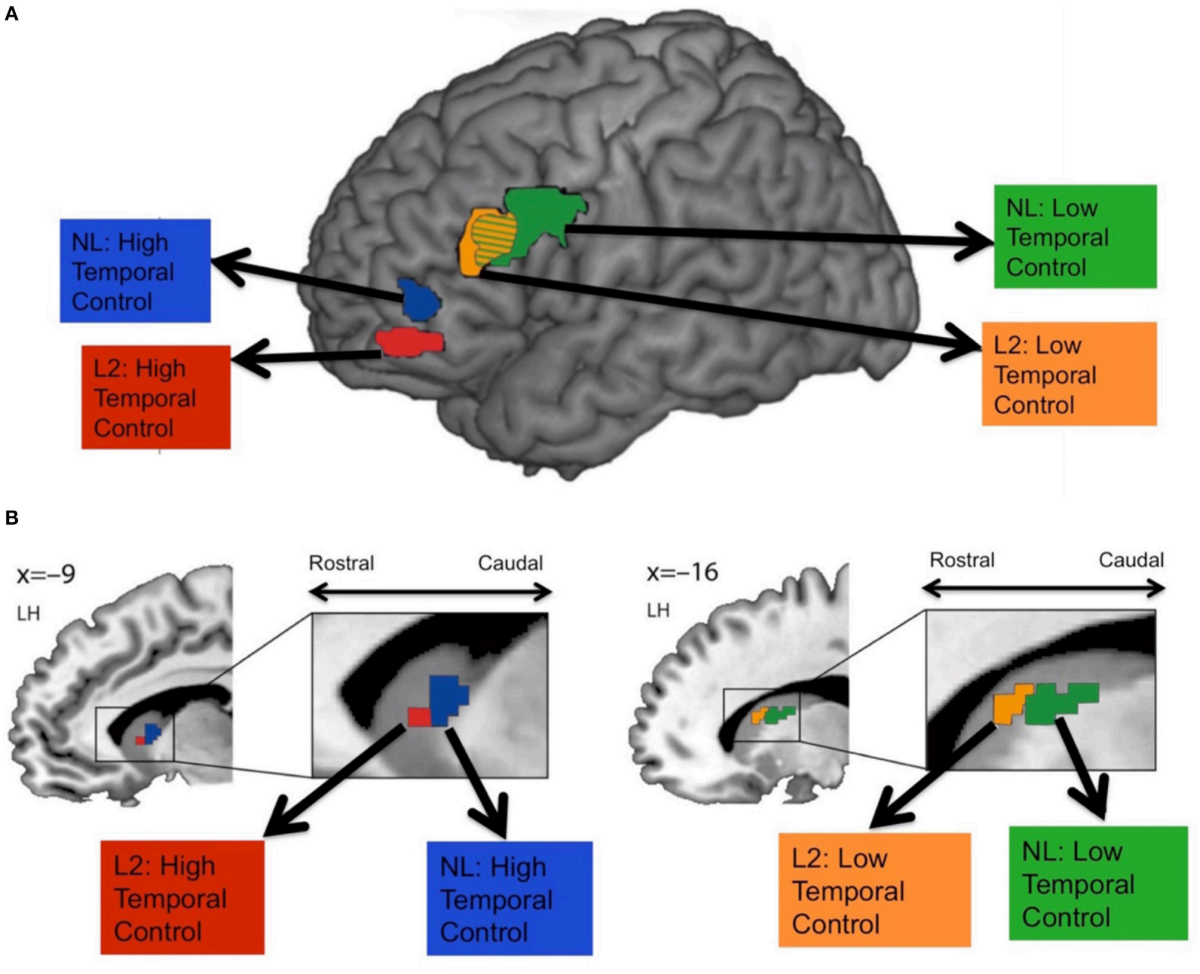
Frontal **(A)** and striatal **(B)** activity during high vs. low temporal control tasks. Participants performed judgments on second language (L2) or a non-existing language (NL) phrases that were temporally close (low temporal control) or temporally more distinct (high temporal control). High temporal control activated more rostral fronto-striatal areas, whereas low temporal control activated more caudal fronto-striatal areas. Figure adapted with permission from the publisher.

Human fMRI studies have also found evidence for a rostral-caudal gradient of fronto-striatal function, which may map onto the anatomical connectivity gradient just presented (Badre and Frank, 2012; Mestres-Missé et al., 2012; Nee and Brown, 2012b; Jeon et al., 2014). For example, Mestres-Missé and colleagues found that higher levels of cognitive control activated both the mid and rostral LPFC (around area 10) as well as the rostral head of the caudate, whereas a mid level of cognitive control activated mid LPFC (around area 46) and the caudal head of the caudate (Mestres-Missé et al., 2012). Combining functional with diffusion weighted MRI, Jeon and colleagues found that a cognitive control task that required judgments about stimuli that were temporally very distinct recruited more rostral LPFC and the rostral caudate, whereas the task that required judgments about stimuli that were temporally less distinct activated more caudal LPFC areas and the caudal caudate (Jeon et al., 2014; see Figure 3). Furthermore, rostral LPFC regions were structurally connected with the rostral caudate whereas caudal LPFC regions were structurally connected with the caudal caudate.

Using fMRI, Nee and Brown (2012b) compared activity patterns during a working memory task requiring holding in mind a higher level context (a higher overall goal that indirectly indicated a later response) vs. a lower level context (updating more concrete information that directly related to a particular type of response). During higher level context processing, rostral LPFC and bilateral basal ganglia were activated, whereas lower level context processing activated caudal LPFC and posterior parietal cortex (PPC). Moreover, rostral LPFC was functionally connected with the basal ganglia during higher level processing, and the caudal LPFC was functionally connected to the PPC during lower level processing. Although these results are not entirely consistent with a rostral-caudal gradient of fronto-striatal function, the type of task used may have influenced the results. For example, Chatham et al. (2014) found that if a context is presented before the trial, fronto-striatal interactions are not as strong as when the context is presented after the trial (Chatham et al., 2014).

Computational models have been put forth to support a hierarchical organization of frontal-striatal loops. For example, a model by Frank and Badre (2012) proposes that hierarchical fronto-striatal loops are organized in a nested manner such that each loop is modulated in a top-down fashion by more rostral loops. For example, the premotor cortex is proposed to be modulated by the pre-premotor cortex, the pre-premotor cortex is modulated by the inferior frontal sulcus, and the inferior frontal sulcus is modulated by the rostral LPFC (Chatham and Badre, 2015). Moreover, it is proposed that the striatum “gates” information into the frontal cortex where that information is maintained (which is referred to as “input gating”) which constrains what type of information is gated to more caudal fronto-striatal areas where, for example, a simple, concrete output can be made, such as a motor response (which is referred to as “output gating”; Frank and Badre, 2012; Chatham et al., 2014). Input gating is proposed to rely less on a hierarchical structure because it only requires information from the striatum being forwarded to the frontal cortex where the information is maintained. In contrast, output gating is proposed to rely more on a hierarchical structure because it allows higher order regions to identify the relevant context, which can then be used to influence lower order regions to determine the correct output (Chatham and Badre, 2015). Computational modeling was found to support these ideas by showing that output gating allows a hierarchical network to obtain a learning advantage over the non-hierarchical network model whereas no such learning advantage is observed during input gating (Frank and Badre, 2012).

## Does dopamine contribute to the hierarchical organization of frontal-striatal loops?

Dopamine is implicated in complex cognitive functions such as working memory and cognitive control (Crofts et al., 2001; for a comprehensive review on the topic see Robbins, 2000; Cools and Robbins, 2004; Robbins and Arnsten, 2009; Ranganath and Jacob, 2015; Cools, 2016; Boot et al., 2017). Given that dopaminergic receptors are found in higher concentrations in frontal cortex and the striatum (Williams and Goldman-Rakic, 1993; Palomero et al., 2015), as compared to most other cortical regions (except for the hippocampus), it is possible that dopamine plays a critical role in the hierarchical organization of fronto-striatal interactions.

**Figure 4:**
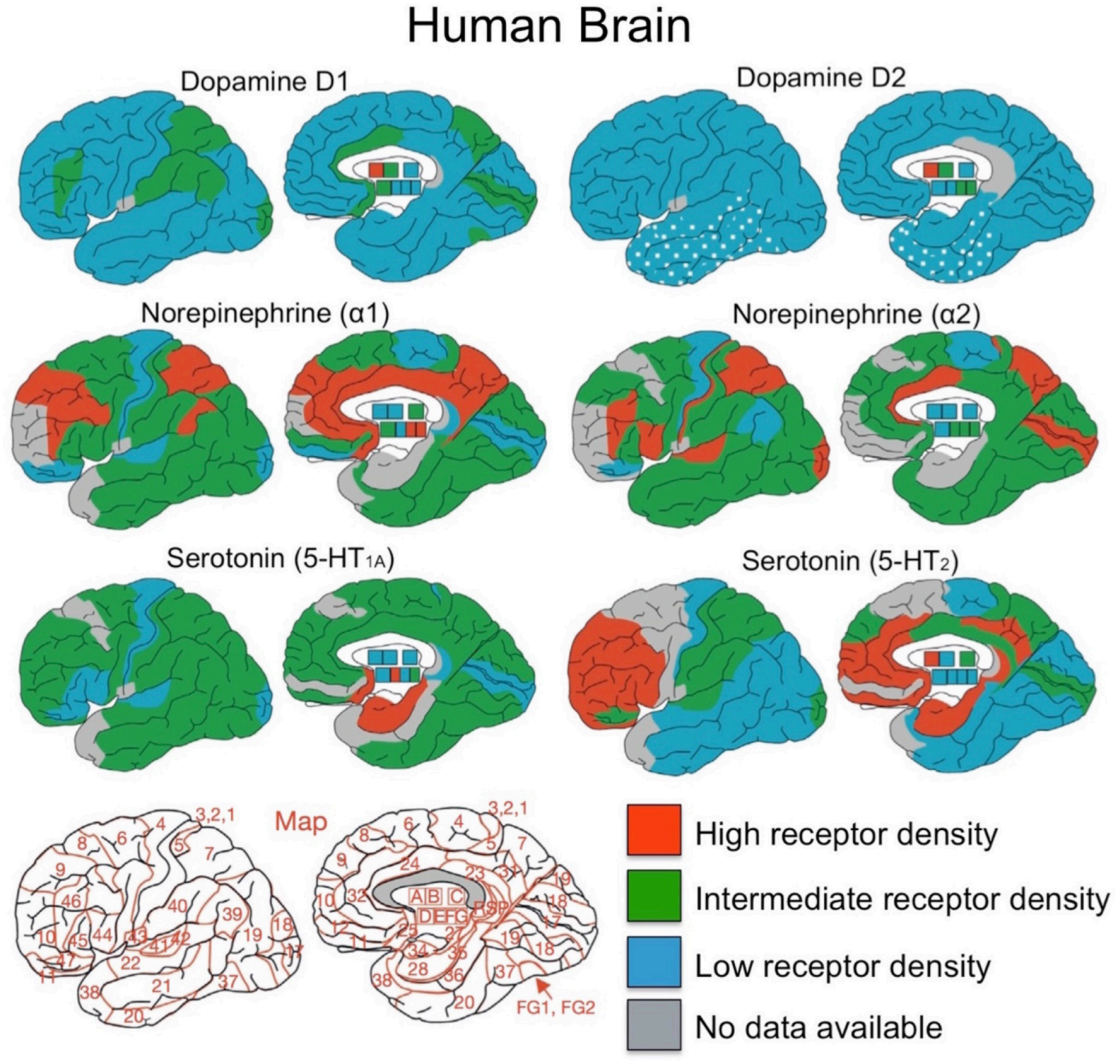
Overview of distributions for dopamine, norepinephrine and serotonin receptors. Brodmann area map is presented in bottom left corner. A, caudate-putamen; B, globus pallidus; C, diencephalon; D, amygdala; E, CA1 region of hippocampus; F, CA2/3 region of hippocampus; G, dentate gyrus. Figure adapted from Palomero et al. (2015) with permission from Elsevier Journals.

What is the regional distribution of dopaminergic receptors in the lateral frontal cortex and the striatum? Although human brain data of this type is limited, Palomero et al. (2015) reviewed published studies with data on neurotransmitter receptor binding in the human brain. Neurotransmitter receptor density maps for 41 Brodmann areas and subcortical nuclei were made, revealing the distribution of glutamate (AMPA, NMDA, Kainate), GABA, acetylcholine, norepinephrine (α1 and α2), serotonin (5-HT1a and 5-HT2) and dopamine (D1 and D2 receptors). The regional distribution of catecholamine receptors - dopamine, norepinephrine and serotonin from these studies are presented in Figure 4.

As can be seen in Figure 4, dopamine has relatively low receptor density throughout the neocortex compared to norepinephrine and serotonin (Palomero et al., 2015; Zilles et al., 2015), yet intermediate concentrations of dopaminergic D1 receptors are present in prefrontal cortex (BA 46), parietal (BA 7, BA 39, and BA 40), the CA1 region of hippocampus, and visual cortex (BA 17). Compared to the D1 receptors, the D2 receptor binding is 2–5 times lower than in cortex, making it difficult to measure in the human brain (Palomero et al., 2015). Dopamine receptor density in the striatum, compared to neocortex and hippocampus, is much higher for both dopaminergic D1 and D2 receptors (Palomero et al., 2015). Also, both caudate and putamen have higher DRD1 and DRD2 receptor densities than in the globus pallidus, which has a nearly five times lower DRD1 receptor density (Sun et al., 2012).

**Figure 5:**
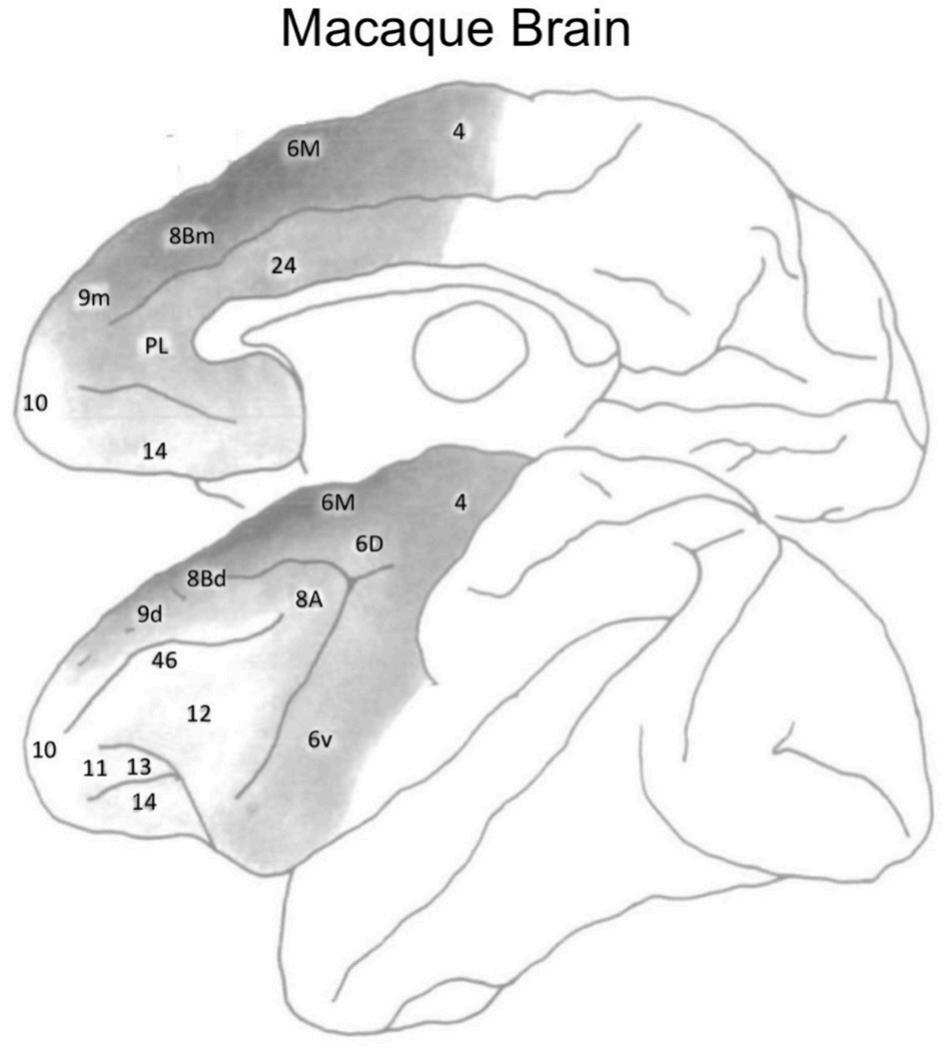
Dopaminergic axon density in the macaque monkey frontal cortex. Darker shades of gray denote a higher density. Figure adapted from Williams and Goldman-Rakic (1993), with permission from the publisher.

In the frontal cortex, Goldman-Rakic and colleagues found regional differences in dopamine receptor density by quantifying dopaminergic axon density in the macaque monkey (Lidow et al., 1991; Williams and Goldman-Rakic, 1993). The highest density of dopaminergic axons was found within medial area 6M and medial area 8Bm (see Figure 5). There is a lower dopaminergic axon density in more rostral frontal regions, as well as in more caudal regions such as primary motor cortex. The lowest dopamine axon density in the frontal cortex is within the most rostral frontopolar regions (see Figure 5). Inspection of Figure 4 suggests that the distribution of dopamine receptors in the human brain differs from that of the macaque monkey. In humans, DRD1 receptor density in frontal cortex is highest in the mid-LPFC (BA46), compared to more rostral and caudal frontal regions. As mentioned previously, DRD2 receptor density is low throughout LPFC.

Human PET imaging (Alakurtti et al., 2013), human post mortem studies (Piggott et al., 1999), and rat immunochemistry studies (Levey et al., 1993) suggest that a rostral-caudal gradient of dopamine receptors may be present in the caudate and putamen. For DRD1 receptors, there is a lower receptor density in caudal, as compared to rostral putamen, but no regional differences exists in the caudate (Piggott et al., 1999). For DRD2 receptors, there is a lower receptor density in caudal, as compared to rostral caudate (Joyce et al., 1991; Levey et al., 1993; Alakurtti et al., 2013; however Piggott et al., 1999 observed the opposite finding). In the putamen, some studies report decreasing DRD2 receptor density from rostral to caudal putamen (Joyce et al., 1991; Levey et al., 1993) whereas others find the opposite (Piggott et al., 1999; Alakurtti et al., 2013). A lack of convergence in these studies may be due to the different methods used (post-mortem tissue vs. *in vivo* PET imaging in humans) and/or different samples used (rats vs. humans).

**Figure 6:**
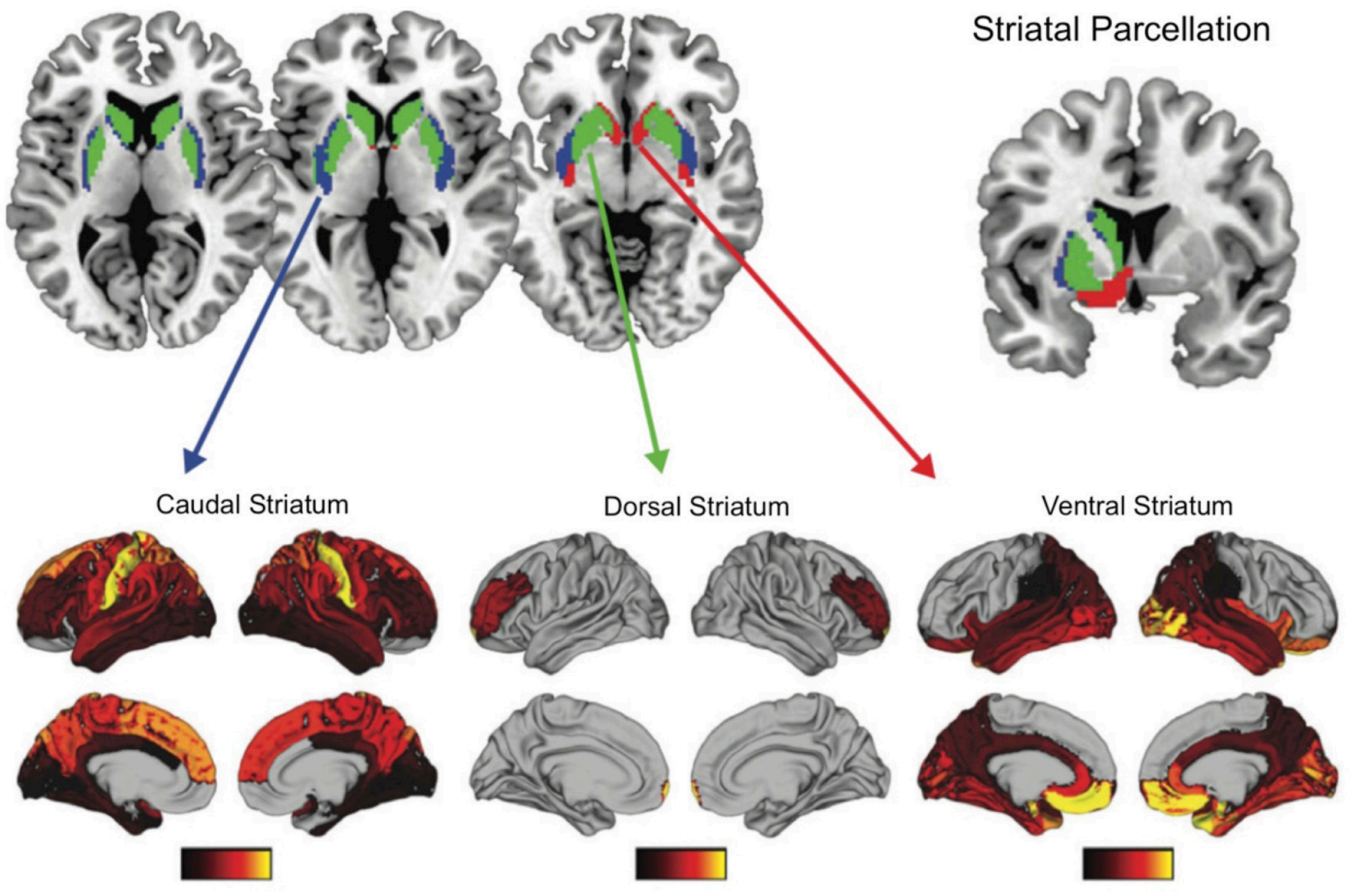
Functional connectivity patterns of three striatal subregions with the rest of the brain. Transcriptional signatures in these subregions is a good predictor of these functional connectivity patterns, with dopamine being an important genetic marker for ventral and dorsal striatum functionally connectivity with limbic regions and LPFC respectively. Figure adapted with permission from Parkes et al. (2017).

What are the implications of regional differences in dopamine receptor distribution in the frontal cortex and striatum? A recent human study, combining diffusion weighted imaging with transcriptonic data, suggests that the distribution of dopaminergic receptors in the striatum maps onto distinct profiles of the structural connectivity of the striatum with the rest of the brain (Parkes et al., 2017). The striatum was parcellated by examining the whole brain structural connectivity, resulting in three distinct striatal regions with unique connectivity with the rest of the brain. The ventral striatum (e.g., nucleus accumbens and ventral putamen) was connected with orbitofrontal, temporal, cingulate areas, hippocampus and amygdala; the dorsal striatum (e.g., head and dorsomedial caudate) was connected mainly with the LPFC, and the caudal striatum (e.g., lateral putamen extending caudally and tail of the caudate) was connected with sensory and motor regions (see Figure 6). Importantly, these structural connectivity patterns related to gene ontology categories such as dopamine signaling and glutamate secretion. The dominant source of genetic variation expressed in the dorsal and ventral striatal connectivity patterns related dopamine signaling whereas the caudal striatum connectivity patterns related to glutamate secretion. These findings suggest that striatal regions that connect to the rostral and mid-LPFC, are dopaminergic rich regions, as compared to more caudal striatal regions.

Based on these and other findings presented thus far in this review, we propose that the regional specificity of dopaminergic receptor distribution in the frontal cortex may contribute to the hierarchical organization of fronto-striatal function. Specifically, we propose that dopaminergic input from the ventral tegmental area (VTA) may be greater in mid-LPFC regions, compared to other frontal regions, given that this region has the highest dopamine receptor distribution in the human brain, compared to more rostral and caudal frontal regions (see Figure 4). As described above, the mid-LPFC region is proposed to be the “top” of a control hierarchy within frontal cortex (Nee and D'Esposito, 2016, 2017) and monkeys (Goulas et al., 2014). Thus, we propose that if dopaminergic input has the greatest influence on frontal-striatal loops at the top of the frontal hierarchy, it will have the greatest influence on all other fronto-striatal loops lower in the hierarchy. This hypothesis can be tested directly in future experiments.

Midbrain neuromodulatory systems other than dopamine, such as norepinephrine and serotonin, may also play a role in the hierarchical organization of lateral frontal cortex. Each of these neurotransmitters have high receptor densities in the frontal cortex (Palomero et al., 2015) and are implicated in higher order cognition (Aston-Jones and Cohen, 2005; Robbins and Arnsten, 2009). Specifically, the highest densities of norepinephrine receptors in frontal areas are within BA 9, 44 and 46 (Palomero et al., 2015). For serotonin, the highest density of 5-HT1A receptors are within BA 9, 10, 46, and 44, whereas the highest density of 5-HT2 receptors are in BA 9, 10, 46, 44, and 45 (Palomero et al., 2015). These regions largely overlap with the dopaminergic receptor distribution in the frontal cortex. Given regional differences in the frontal density of dopamine, norepinephrine, and serotonin in LPFC, each transmitter may make different contributions to frontal cortex function. For example, the distribution of serotonin receptors appears more widespread throughout the frontal cortex, whereas dopamine and norepinephrine appear to have their highest concentration within mid lateral PFC (e.g., BA 46). These differences in the regional distribution of receptors suggest that dopamine and norepinephrine may play a greater role than serotonin in the hierarchical organization of frontal cortex. It will be important for future studies to test this hypothesis, as well as uncover the differential contribution of dopamine vs. norepinephrine.

## Conclusions and future directions

We have reviewed functional MRI, neurophysiological and neuropsychological evidence supporting a hierarchical organization of frontal cortex as well as the evidence that a rostral-caudal gradient may exist in the anatomical and functional organization of fronto-striatal loops, as well as in the distribution of dopamine receptors. Finally, we have put forth the hypothesis that regional differences in dopamine receptors may lead to modulation of specific fronto-striatal loops more than others, which can contribute to the hierarchical organization of these loops. Specifically, the evidence we reviewed suggests that midbrain dopaminergic projections may be greatest in a mid-LPFC region, which in turn could result in this region having the greatest influence on the striatum. Future experiments, combining PET imaging with fMRI, could more firmly establish the link between dopamine receptor density in frontal cortex and striatum and the organization of frontal-striatal loops. Furthermore, advanced fMRI analytic methods such as granger causality or dynamic causal modeling could also test whether the mid-LPFC region with the highest dopamine receptor density has the greatest influence over other frontal regions and striatum. Such indirect evidence from PET and fMRI studies should be supplemented with causal studies (e.g., patients with frontal lesions, or TMS disruption of frontal cortex in healthy individuals).

## Author contributions

DV and MD conceived the concept of the review. DV and MD wrote the paper.

### Conflict of interest statement

The authors declare that the research was conducted in the absence of any commercial or financial relationships that could be construed as a potential conflict of interest.
